# Supplementary material for: Cell type-specific contributions to a persistent aggressive internal state in female Drosophila
Source: eLife. 2025 Jul 25;12:RP88598. doi: 10.7554/eLife.88598 (PMC12296262; doi:10.7554/eLife.88598)
Supplement: Supplementary file 2. [file elife-88598-supp2.docx]

**Sample size and Statistics**

**Figure 2B – C**

Raster plots – (B) Each instance of head butting and shoving for both flies in the trial following the removal of the barrier.

Analysis – (C) Average fraction of time spent performing head butting and shoving behavior over each of five 2-minute periods: 11 – 13, 13 – 15, 15 – 17, 17 – 19, and 19 – 21 minutes after the stimulus.

Three biological repeats were performed, and the data was combined.

| Figure | Condition | # | Sample Size | Statistical  Test | Comparison | P-value |
| --- | --- | --- | --- | --- | --- | --- |
| 2C | Cntrl>CsChrimson | 1 | 23 | Kruskal-Wallis | 11 – 13 min: |  |
|  | pC1d>CsChrimson  aIPg>CsChrimson | 2  3 | 23  20 | (P = 0.0002) | 1 v 2  1 v 3 | >0.9999  0.0048 |
|  | pC1d+e>CsChrimson | 4 | 25 | Kruskal-Wallis | 1 v 4  13 – 15 min: | 0.0005 |
|  |  |  |  | (P = 0.0029)  Kruskal-Wallis  (P = 0.1368)  Kruskal-Wallis  (P = 0.2928)  Kruskal-Wallis  (P = 0.3120) | 1 v 2  1 v 3  1 v 4  15 – 17 min:  1 v 2  1 v 3  1 v 4  17 – 19 min:  1 v 2  1 v 3  1 v 4  19 – 21 min:  1 v 2  1 v 3  1 v 4 | >0.9999  0.0636  0.0166  >0.9999  0.8313  0.2141  >0.9999  0.5071  0.1960  0.5090  >0.9999  >0.9999 |
|  |  |  |  |  |  |  |

**Figure 2 – Figure Supplement 1**

Raster plots – Each instance of head butting and shoving for only the tester flies in the trial.

Analysis – Average fraction of time the tester fly spent performing head butting and shoving behavior over each of three periods: during, immediately following (0 – 45 seconds), and 45 – 105 seconds after the stimulus.

Three biological repeats were performed, and the data was combined.

| Figure | Condition | # | Sample Size | Statistical  Test | Comparison | P-value |
| --- | --- | --- | --- | --- | --- | --- |
| 2.1 | Cntrl>CsChrimson | 1 | 25 | Kruskal-Wallis | During: |  |
|  | pC1d>CsChrimson  aIPg>CsChrimson | 2  3 | 26  41 | (P<0.0001) | 1 v 2  1 v 3 | <0.0001  0.0349 |
|  | pC1d+e>CsChrimson | 4 | 28 | Kruskal-Wallis | 1 v 4  0 – 45 sec: | <0.0001 |
|  |  |  |  | (P<0.0001)  Kruskal-Wallis  (P = 0.0003) | 1 v 2  1 v 3  1 v 4  45 – 105 min:  1 v 2  1 v 3  1 v 4 | 0.0760  <0.0001  0.0037  0.6675  0.0002  0.3495 |
|  |  |  |  |  |  |  |

**Figure 3 – Figure Supplement 1**

Analysis – Average fraction of time spent performing head butting and shoving behavior over each of four periods: prior, during, immediately following (0 – 30 seconds), and 30 – 60 seconds after the stimulus.

Three biological repeats were performed, and the data was combined.

| Figure | Condition | # | Sample Size | Statistical  Test | Comparison | P-value |
| --- | --- | --- | --- | --- | --- | --- |
| 3.1 | Cntrl>Kir2.1 | 1 | 16 | Mann-Whitney | Prior: |  |
|  | pC1d>Kir2.1 | 2 | 15 | Mann-Whitney | 1 v 2  During: | 0.3729 |
|  |  |  |  | Mann-Whitney  Mann-Whitney | 1 v 2  0 – 30 s:  1 v 2  30 – 60 s:  1 v 2 | 0.2886  0.2874  0.2011 |
|  |  |  |  |  |  |  |

**Figure 4**

Analysis – Average area under curve value over the 10-second window in the baseline (5-15 seconds), stimulation (39-49 seconds), and post-stimulation (start at 3 decay time constant after stimulation).

Three to four biological repeats were performed, and the data was combined.

| Figure | Condition | # | Sample Size | Statistical  Test | Comparison | P-value |
| --- | --- | --- | --- | --- | --- | --- |
| 4A | pC1d>Chrimson pC1d>GCaMP  Baseline  pC1d>Chrimson pC1d>GCaMP  Stim  pC1d>Chrimson pC1d>GCaMP  Post-Stim | 1  2  3 | 4 | Paired t-test  Paired t-test  Paired t-test | 1 v 2  2 v 3  1 v 3 | 0.0012  0.0025  0.0426 |
|  |  |  |  |  |  |  |

| Figure | Condition | # | Sample Size | Statistical  Test | Comparison | P-value |
| --- | --- | --- | --- | --- | --- | --- |
| 4B | aIPg>Chrimson aIPg>GCaMP  Baseline  aIPg>Chrimson aIPg>GCaMP  Stim  aIPg>Chrimson aIPg>GCaMP  Post-Stim | 1  2  3 | 3 | Paired t-test  Paired t-test  Paired t-test | 1 v 2  2 v 3  1 v 3 | 0.0003  0.0075  0.1352 |
|  |  |  |  |  |  |  |

| Figure | Condition | # | Sample Size | Statistical  Test | Comparison | P-value |
| --- | --- | --- | --- | --- | --- | --- |
| 4C | pC1d>Chrimson aIPg>GCaMP  Baseline  pC1d>Chrimson aIPg>GCaMP  Stim  pC1d>Chrimson aIPg>GCaMP  Post-Stim | 1  2  3 | 3 | Paired t-test  Paired t-test  Paired t-test | 1 v 2  2 v 3  1 v 3 | 0.0359  0.0212  0.1948 |
|  |  |  |  |  |  |  |

| Figure | Condition | # | Sample Size | Statistical  Test | Comparison | P-value |
| --- | --- | --- | --- | --- | --- | --- |
| 4D | aIPg>Chrimson pC1d>GCaMP  Baseline  aIPg>Chrimson pC1d>GCaMP  Stim  aIPg>Chrimson pC1d>GCaMP  Post-Stim | 1  2  3 | 3 | Paired t-test  Paired t-test  Paired t-test | 1 v 2  2 v 3  1 v 3 | 0.0893  0.4300  0.2089 |
|  |  |  |  |  |  |  |

**Figure 4 – Figure Supplement 1**

Analysis – Average area under curve value over the 10-second window in the baseline (5-15 seconds), stimulation (309-319 seconds), and post-stimulation (start at 3 decay time constant after stimulation).

Three biological repeats were performed, and the data was combined.

| Figure | Condition | # | Sample Size | Statistical  Test | Comparison | P-value |
| --- | --- | --- | --- | --- | --- | --- |
| 4.1A | pC1d>Chrimson pC1d>GCaMP  Baseline  pC1d>Chrimson pC1d>GCaMP  Stim  pC1d>Chrimson pC1d>GCaMP  Post-Stim | 1  2  3 | 3 | Paired t-test  Paired t-test  Paired t-test | 1 v 2  2 v 3  1 v 3 | 0.0016  0.0014  0.0349 |
|  |  |  |  |  |  |  |

| Figure | Condition | # | Sample Size | Statistical  Test | Comparison | P-value |
| --- | --- | --- | --- | --- | --- | --- |
| 4.1B | aIPg>Chrimson aIPg>GCaMP  Baseline  aIPg>Chrimson aIPg>GCaMP  Stim  aIPg>Chrimson aIPg>GCaMP  Post-Stim | 1  2  3 | 3 | Paired t-test  Paired t-test  Paired t-test | 1 v 2  2 v 3  1 v 3 | 0.0004  0.0241  0.2100 |
|  |  |  |  |  |  |  |

| Figure | Condition | # | Sample Size | Statistical  Test | Comparison | P-value |
| --- | --- | --- | --- | --- | --- | --- |
| 4.1C | pC1d>Chrimson aIPg>GCaMP  Baseline  pC1d>Chrimson aIPg>GCaMP  Stim  pC1d>Chrimson aIPg>GCaMP  Post-Stim | 1  2  3 | 3 | Paired t-test  Paired t-test  Paired t-test | 1 v 2  2 v 3  1 v 3 | 0.0172  0.0212  0.2388 |
|  |  |  |  |  |  |  |

| Figure | Condition | # | Sample Size | Statistical  Test | Comparison | P-value |
| --- | --- | --- | --- | --- | --- | --- |
| 4.1D | aIPg>Chrimson pC1d>GCaMP  Baseline  aIPg>Chrimson pC1d>GCaMP  Stim  aIPg>Chrimson pC1d>GCaMP  Post-Stim | 1  2  3 | 3 | Paired t-test  Paired t-test  Paired t-test | 1 v 2  2 v 3  1 v 3 | 0.2294  0.3480  0.1675 |
|  |  |  |  |  |  |  |
